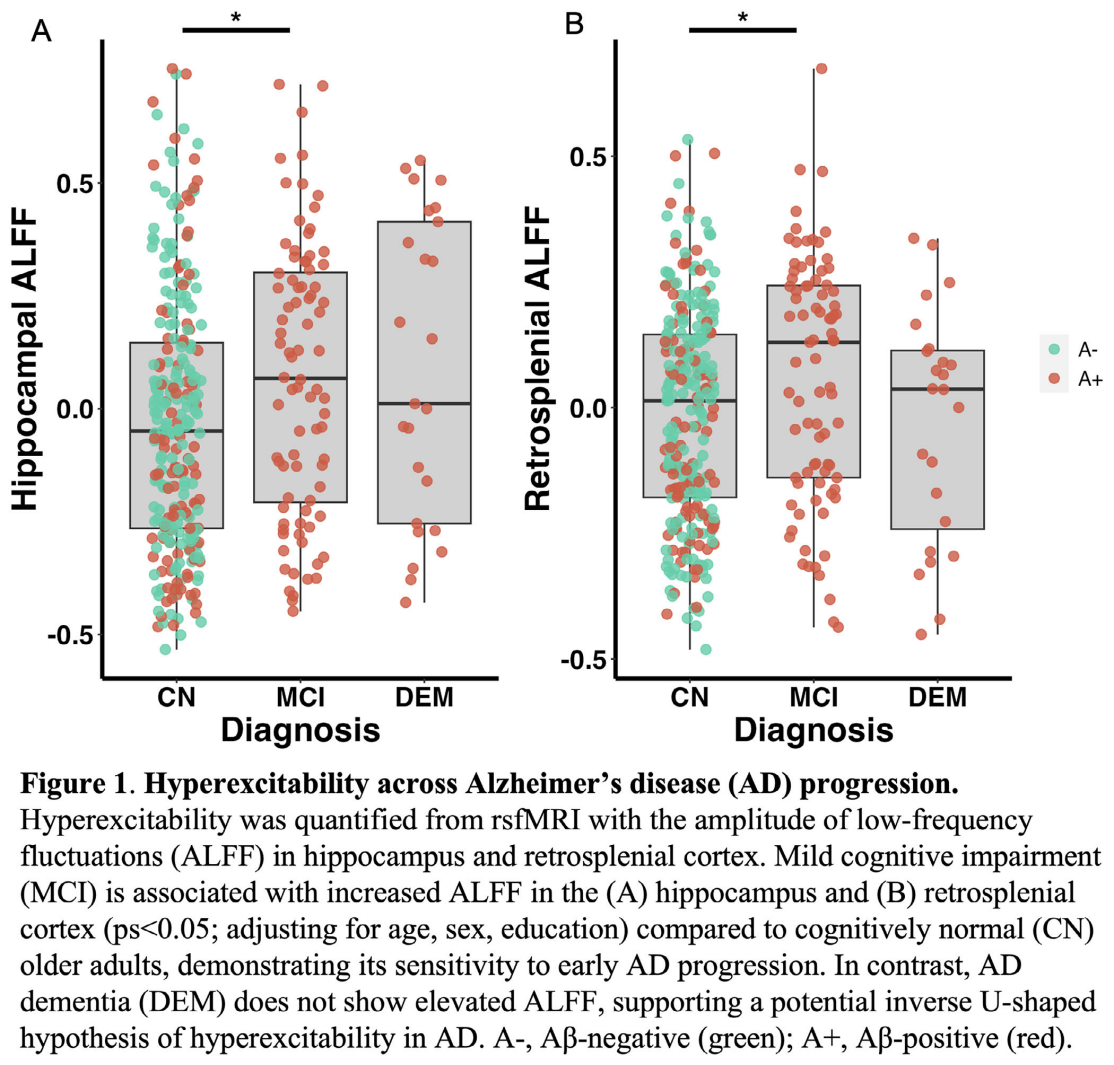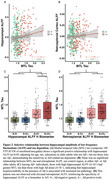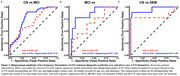# Investigating hippocampal hyperexcitability as a biomarker of Alzheimer’s disease

**DOI:** 10.1002/alz70862_110095

**Published:** 2025-12-23

**Authors:** Casey R Vanderlip, Jenna N. Adams

**Affiliations:** ^1^ University of California, Irvine, Irvine, CA USA; ^2^ University of California, Irvine, CA USA

## Abstract

**Background:**

Hippocampal hyperexcitability is an early feature of Alzheimer’s disease (AD) that may drive pathology. However, current models of AD risk focusing on the A/T/N framework (Aβ /Tau/Neurodegeneration) do not include hyperexcitability as a key disease biomarker. Here, we investigated a candidate hyperexcitability (“H”) biomarker from resting‐state fMRI (rsfMRI) ‐ the amplitude of low‐frequency fluctuations (ALFF), which reflects the intensity of spontaneous brain activity – to determine if it provides sensitive information about AD progression.

**Method:**

We analyzed 386 older adults spanning the AD spectrum (277 cognitively normal, CN; 84 Aβ+ mild cognitive impairment, MCI; 25 Aβ+ AD dementia) from ADNI who underwent rsfMRI, Aβ‐PET (18F‐FBP/18F‐FBB), and tau‐PET (18F‐FTP) within a year. ALFF was quantified from rsfMRI in the hippocampus as the primary “H” candidate, as well as the retrosplenial cortex, which served as a control region. Aβ‐PET (18F‐florbetapir or 18F‐Florbetaben) global Centiloids (CL) and Aβ+ status (>20 CL), tau‐PET (18F‐Flortaucipr) in the medial temporal lobe (MTL) composite (entorhinal and amygdala mean SUVR), and hippocampal volume were used as A/T/N biomarkers, respectively. Relationships between ALFF, A/T/N biomarkers, and diagnosis were examined, controlling for age, sex, and education.

**Result:**

Hippocampal and retrosplenial ALFF were elevated in individuals with MCI compared to CN individuals (Figure 1). Higher hippocampal ALFF significantly correlated with increased MTL tau in Aβ+ individuals, whereas there was no relationship with retrosplenial ALFF (Figure 2A‐B). Further, among Aβ+ individuals, those with high hippocampal ALFF (A+H+) had greater MTL tau than those with high Aβ alone (A+H‐), demonstrating hippocampal hyperexcitability in the context of Aβ is related to elevated tau pathology (Figure 2C). Finally, ROC analyses revealed that adding hippocampal ALFF to a basic A/T/N biomarker model improved diagnostic discrimination (Figure 3), suggesting hippocampal ALFF captures cognitive impairment beyond the A/T/N framework.

**Conclusion:**

These findings indicate that hippocampal ALFF, a proxy of hippocampal hyperexcitability, serves as a valuable biomarker of AD. Planned analyses include assessing the sensitivity of hippocampal ALFF for longitudinal prediction of AD pathology and phenoconversion from CN to MCI or dementia. Future frameworks of AD should incorporate “H” biomarkers of hyperexcitability to further understand disease progression and risk.